# Conventional ultrasonography and elastosonography in diagnosis of malignant thyroid nodules: A systematic review and meta-analysis

**DOI:** 10.3389/fendo.2022.1082881

**Published:** 2023-01-06

**Authors:** Dian Zhang, Xiao-Na Wang, Li Jiang, Chun-Xia Yu, Yue-Nan Chen, Xue-Juan Yu, Mei-Fang Pan

**Affiliations:** Department of Ultrasound, Xiangcheng People’s Hospital, Suzhou, Jiangsu, China

**Keywords:** thyroid nodules, conventional ultrasonography (US), elastosonography, meta-analysis, malignant

## Abstract

**Purpose:**

To evaluate the diagnostic value of conventional ultrasound and elastosonography in malignant thyroid nodules by meta-analysis.

**Methods:**

The literature included in the Cochrane Library, PubMed, and Embase were searched by using “elastosonography, ultrasonography, thyroid nodules” as the keywords. The clinical studies using elastosonography and conventional ultrasound to diagnose thyroid nodules were selected, and histopathology of thyroid nodules was used as reference standards. The quality evaluation and heterogeneity test were performed on the literature that met the requirements, the combined specificity and sensitivity were pooled, and a comprehensive ROC curve analysis was performed. The Quality Assessment of Diagnostic Accuracy Studies (QUADAS) tool was utilized to evaluate the quality of each included study. Meta-DiSc version 1.4, StataSE 12 and Review Manager 5.4 were used.

**Results:**

A total of nine studies assessed 3066 thyroid nodules (2043 benign and 1023 malignant). The pooled sensitivity, specificity, PLR, NLR, and DOR of conventional ultrasound for the diagnose of malignant thyroid nodules were 0.833 (95% CI 0.809-0.855), 0.818 (95% CI 0.801-0.835), 4.85 (95% CI 4.36-5.39), 0.20 (95% CI 0.17-0.23), and 29.38 (95% CI 23.28-37.08), respectively, with an AUC of 0.9068. Also, the pooled sensitivity, specificity, PLR, NLR, and DOR of elastosonography were 0.774 (95% CI 0.741-0.804), 0.737 (95% CI 0.715-0.758), 3.14(95% CI 2.85-3.47), 0.29 (95% CI 0.25-0.34), and 9.35 (95% CI 7.63-11.46), respectively, with an AUC of 0.8801. Three studies provided data regarding the conventional ultrasound and elastosonography. The pooled sensitivity, specificity, PLR, NLR, DOR, and AUC were 0.902 (95% CI 0.870-0.928), 0.649 (95% CI 0.616-0.681), 2.72 (95% CI 2.46-3.00), 0.14 (95% CI 0.11-0.19), 25.51 (95%CI 17.11–38.03), and 0.9294.

**Conclusion:**

The existing evidence shows that elastosonography cannot completely replace conventional ultrasound in the diagnosis of malignant thyroid nodules, and the combination of elastosonography and conventional ultrasound gives a better diagnostic precision.

**Systematic review registration:**

www.crd.york.ac.uk, identifier PROSPERO CRD42022375808.

## 1 Introduction

The incidence of thyroid nodules is high, 68% of the normal population can be detected, and malignant nodules account for 5-10% (1). Ultrasound has a high value for its detection and diagnosis (1). With the application of high-resolution ultrasound, the detection of thyroid nodules has gradually increased over recent years. Various thyroid imaging reporting and data systems for US features can be used to improve the diagnostic accuracy of thyroid nodules (2, 3). In 2005, Lyshchik (4) reported thyroid elastography for the first time. At present, elastosonography (ES) has been gradually applied to the diagnosis of thyroid diseases. ES uses the different elastic coefficients between the tumor or other diseased area and the surrounding normal tissue, resulting in different strain sizes, and is displayed in color coding to judge the elasticity of the diseased tissue (5). The elasticity coefficient of thyroid cancer is greater than that of normal glands or benign lesions, which can be used for differential diagnosis of benign and malignant thyroid tumors. Nevertheless, it has been reported that ultrasound is characterized by high sensitivity and low specificity (6, 7). If benign nodules and malignant nodules appear in the same image, the two images are easily overlapped, resulting in misdiagnosed (8). Currently, ultrasound-guided fine-needle aspiration biopsy (US-FNAB), due to its low cost and ease of operation, is the cornerstone for the evaluation of thyroid nodular lesions (9–11). However, there is a possibility of bleeding and hematoma formation during biopsy (12). Therefore, how to reduce the incidence of misdiagnosis by ultrasound technology is of great significance to clinical treatment. In this study, Meta-analysis was used to systematically and quantitatively evaluate the literature on the diagnosis of thyroid nodules by ES and conventional ultrasonography (CUS), to provide more objective evidence-based medical evidence for clinical practice.

## 2 Materials and methods

This systematic review and meta-analysis were performed following the Preferred Reporting Items for Systematic Reviews and Meta-Analyses (PRISMA) guidelines (13).

### 2.1 Search strategy

PubMed, Cochrane library, and Embase were searched, and the search strategy was: ((conventional ultrasound) AND (ultrasound elastography)) AND (thyroid nodules). The search time was from the start of the library building to September 2022. Two investigators (DZ and XW) searched online to obtain the original data, and the reference lists of all relevant articles were also scanned. The retrieved literature includes conference papers and dissertations. A combination of subject headings and free words, manual search, and network search was used to search the references of the included literature for a second time. All retrieved citations were exported to Zotero and checked for duplicates.

### 2.2 Inclusion and exclusion criteria

Literature screening were carried out according to the inclusion criteria for diagnostic test studies in the Screening and Diagnostic Test Methods Group of the Cochrane Collaboration. The inclusion criteria of this study were as follows: (1) English literature; (2) No significant differences in age of study subjects; (3) Detailed description of each technique; (4) Both examinations are in the same group of cases; (5) The research subjects include a variety of cases of thyroid sarcoidosis, which can represent the total number of thyroid nodules cases; (6) histopathology examination results; (7) The data in the four-table table can be obtained directly or indirectly; (8) The elastography is based on the 4-point method or the 5-point method. Exclusion criteria: (1) Abstracts, reviews, reviews, or case reports; (2) Repeated publication of data; (3) Incomplete original data; (4) ES and CUS studies did not involve the same group of cases.

### 2.3 Data extraction

Data extraction from the literature was performed by two investigators alone (DZ and XW). The following data were extracted: first author, publication year, country of origin, type of study, number of patients, sex, number of lesions, mean age, lesion size, number of malignant lesions, instruments, system parameters, and diagnostic criteria. After extraction, the data were cross-checked, and if different, they were passed to a third investigator for verification.

### 2.4 Regression covariates and settings

Regression covariates and settings are set as follows:(1) Region: 1 for Asia, 0 for non-Asia. (2) Study type: 1 for retrospective studies and 0 for other studies. (3) ES evaluation criteria: 0 for 5-point scale and 1 for a 4-point scale.; (4) CUS evaluation criteria: 6 signs of echogenicity, microcalcifications, margins, shape, blood flow, and posterior echo are pointed out in the literature as 1, and 0 if not pointed out.

### 2.5 Literature quality assessment

Following QUADAS recommended by the Cochrane Collaboration as a quality assessment tool for diagnostic tests, we also used nine additional quality assessment questions proposed by the Cochrane Diagnostic Test Accuracy Working Group (14). According to the specific content of each item, the answer options are divided into three situations: “Yes”, “No” and “Uncertain”. We did not calculate an overall score to estimate the overall quality of each study. The methodological quality assessment was performed by two independent reviewers who resolved disagreements by discussing the cases and reaching a consensus. Literature was screened, data extracted, and cross-checked according to pre-established inclusion and exclusion criteria.

### 2.6 Statistical analysis

Meta-DiSc version 1.4 software was used for meta-analysis and SROC curve was constructed. Graphs summarizing methodological quality and risk of bias were created using Review Manager (RevMan, version 5.4, Cochrane IMS). At the same time, analyze the heterogeneity of the diagnostic odds ratio (DOR) of each study. If I^2^<50%, there was no heterogeneity, and the fixed effect model (Mantel–Haenszel method) was used for analysis; if I^2^>50%, there was heterogeneity, and random effects (DerSimonian–Laird method) were used as analysis model. The sensitivity analysis was carried out by StataSE 12 (Stata Corporation, College Station, TX). Meta-analysis was then performed on all included studies, and the combined sensitivity, specificity, positive likelihood ratio (PLR), negative likelihood ratio (NLR), and area under the comprehensive receiver operating characteristic (ROC) curve were calculated. All results were expressed with 95% CI, and P<0.05 indicated a statistically significant difference.

## 3 Results

### 3.1 Overall characteristics of selected studies

**Figure 1:**
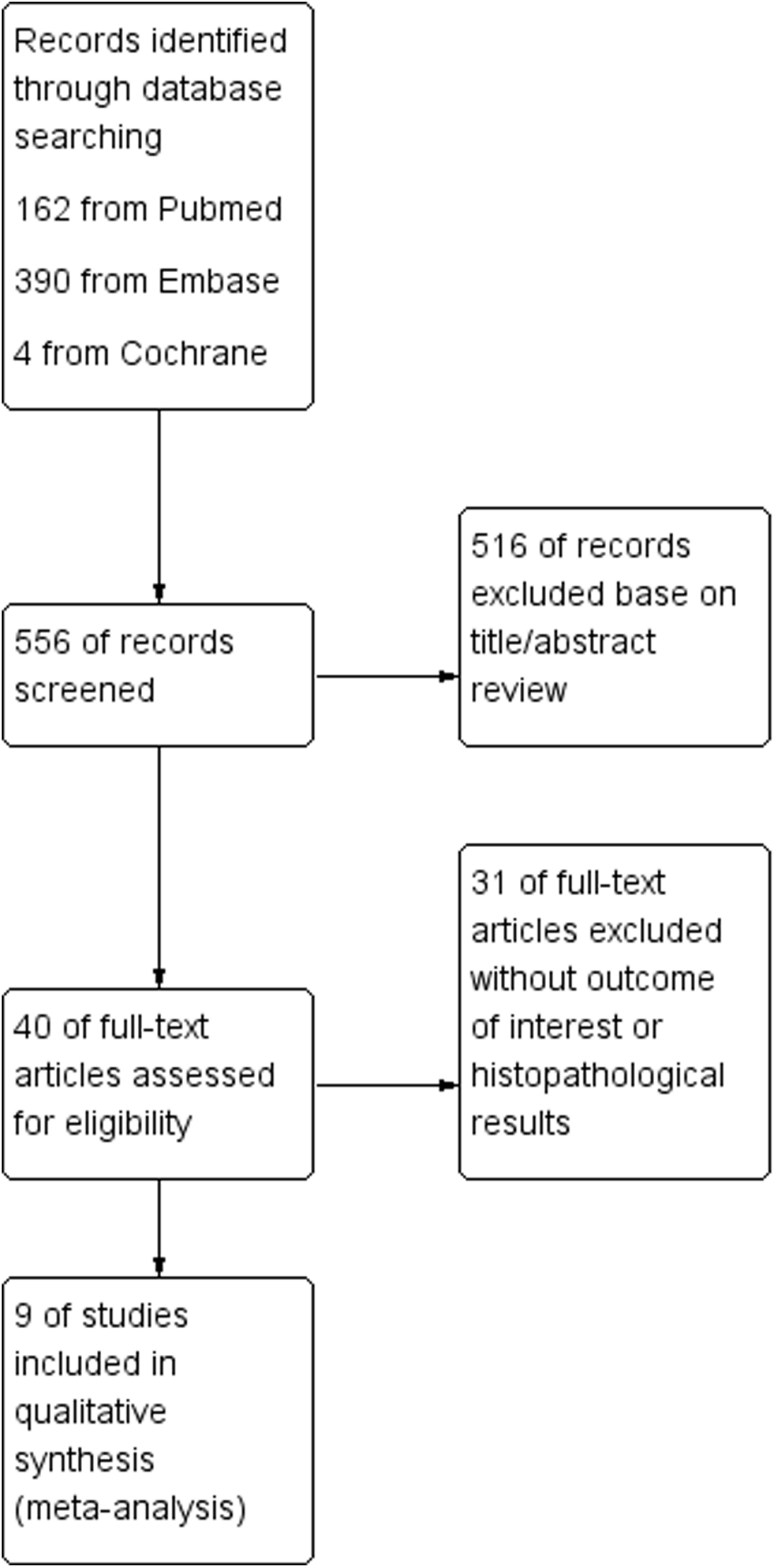
Flowchart of the study selection process.

As shown in **Figure 1**, a total of 556 published articles (162 from PubMed, 390 from Embase, and 4 from Cochrane) were identified. First, after reviewing the titles and abstracts of the articles, we excluded 516 irrelevant articles. A secondary screening of the remaining 40 articles was performed to exclude 31 articles without interest or histopathological results. Finally, nine full-text articles were identified as eligible for the meta-analysis (15–23). **Figure 1** illustrates the steps of the literature search process.

### 3.2 Characteristics of the included studies

The characteristics of all included studies are shown in **Table 1**. The included articles were published between 2012 and 2020 and evaluated a total of 3066 thyroid nodules (2043 benign and 1023 malignant) in 2470 patients. Three studies included more than 500 lesions (19, 20, 22), and one studies included less than 100 lesions (23). Lesions with diameters ranging from 0.3 to 9.3 cm. In terms of study site distribution, 1 were from Europe, 7 from Asia and 1 from Africa. In three studies (19, 22, 23), a combination of both methods was used. The assessment methods and classifications of resilience scores vary among individual studies. Six studies used a 4-point scale and three studies used a 5-point scale.

**Table 1:** Summary of included study characteristics.

Author (Year)	Country	Design	Patients (nodules)	Sex (M/F)	Age in years(mean ± SD)	Nodule size (cm)	Malignant nodules (n)	Ultrasound system	Probe frequency (MHz)
Shuzhen (2012) (15)	China	NA	244(291)	61/183	43.38 ± 0.83	0.3-3.2	66	Hitachi HV-900	6-13
					(Range 7-79)				
Yang (2017) (16)	China	retrospective study	123(150)	22/101	40	0.5-1.6	50	EUB-7500	6-13
					(Range 23-56)				
Reginelli (2014) (17)	Italy	prospective study	354(493)	90/264	41.2 ± 9.2	NA	71	LOGIQ 9 system	NA
					(Range 18-73)				
Zhao (2020) (18)	China	retrospective study	174(177)	44/130	45	0.4-9.3	81	Phillips iu22 scanner and HITACHI Vision 900 system	5-12
					(Range 22-75)				
Moon (2012) (19)	Korea	retrospective study	676(703)	120/556	49.7	0.5-1	217	EUB-7500	6-14
					(Range 18-79)				
Wu (2020) (20)	China	retrospective study	458(557)	112/346	46.6	0.5-4.9	286	Hitachi HV-900 or Avius	7.5-13
					(Range 21-82)				
Garg (2018) (21)	India	NA	97(117)	NA	43	3.4-6	33	ACUSON S2000	7.5-12
					(Range 31-52)				
Shao (2015) (22)	China	NA	297(512)	66/231	42.15( ± 11.35)	NA	203	GE Vivid E9 ultrasound system	6-15
Shweel (2013) (23)	Egypt	prospective study	47(66)	12/35	41 ± 11	NA	16	ACUSON S2000	7.5-13
					(Range 22-70)				

### 3.3 Assessment of methodologic quality

**Figure 2:**
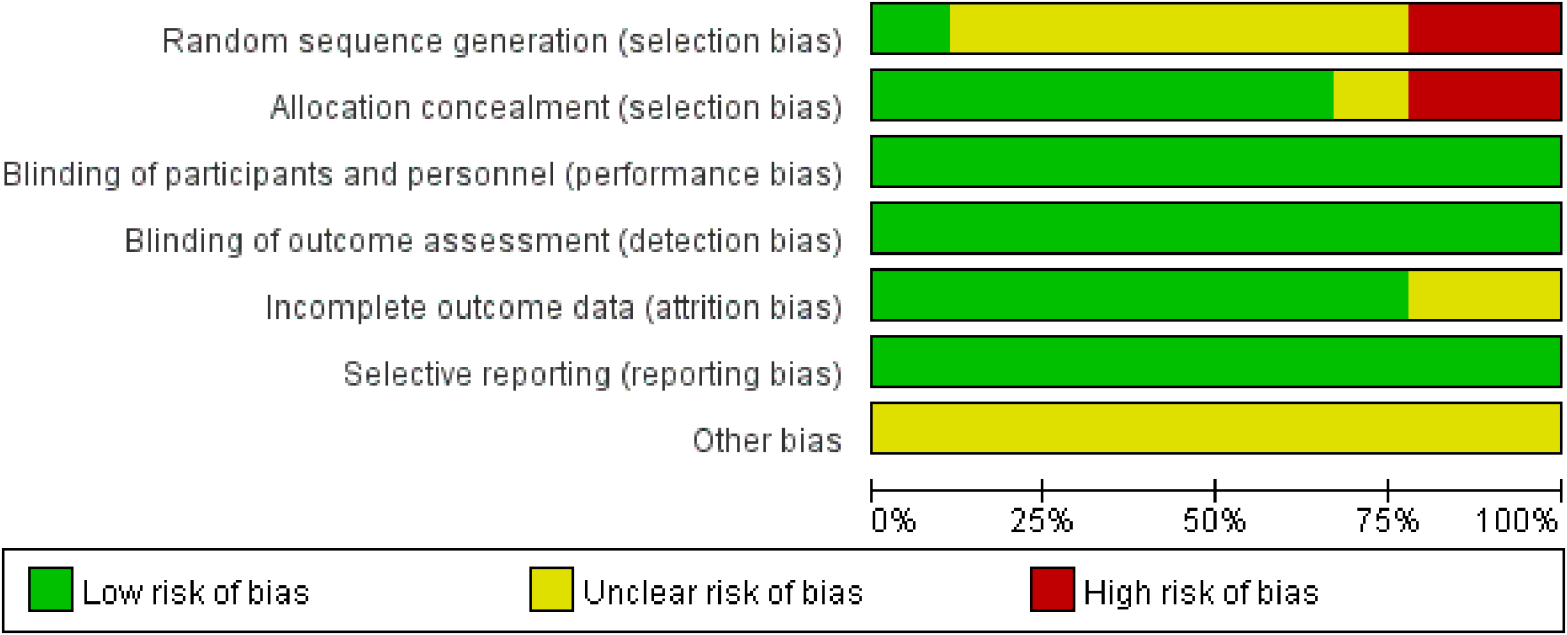
Percentage of included studies with the risk of bias.

**Figure 3:**
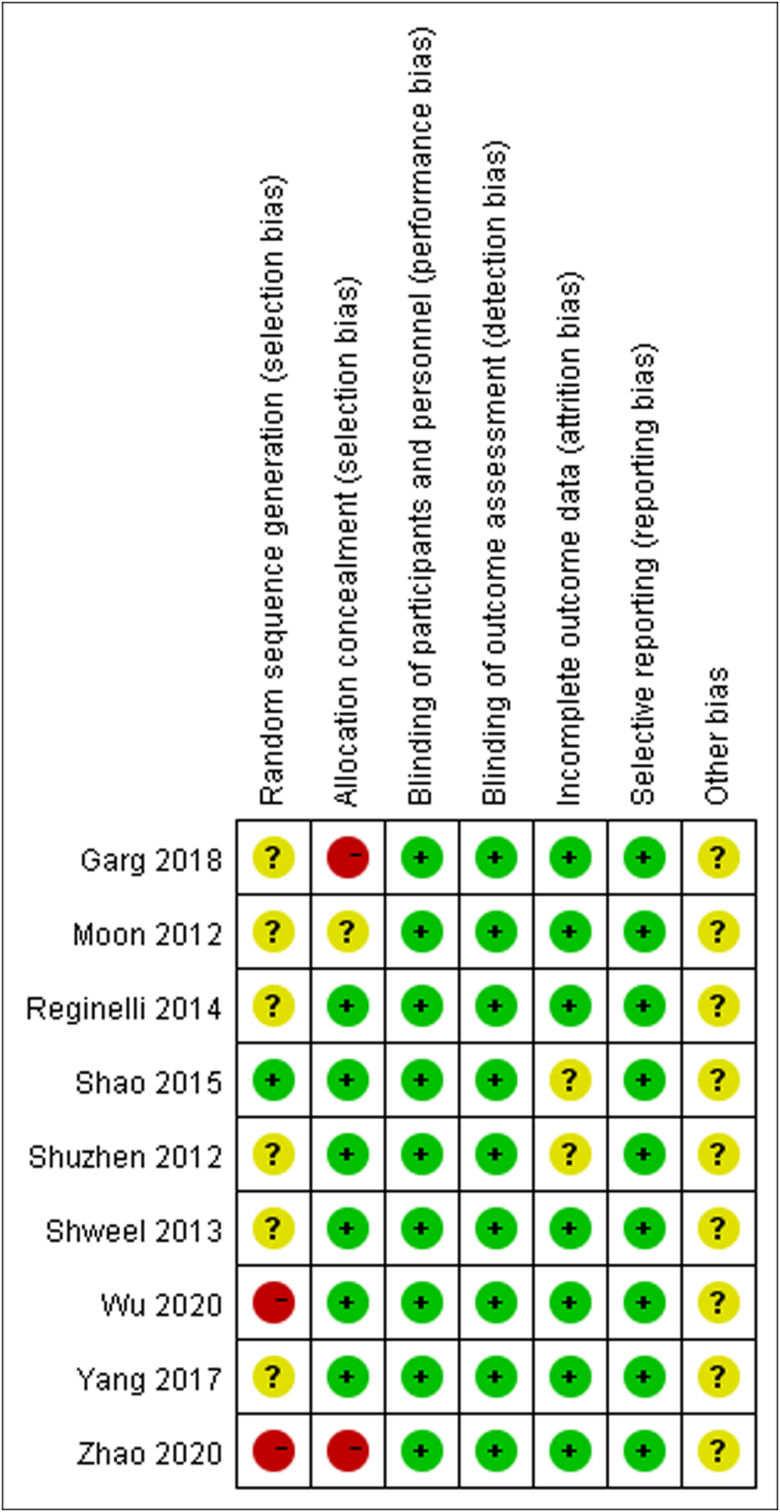
The assessment of risk of bias for included study. Quality is represented by colors using green (+) as yes (high quality), yellow (?) as unclear, and red (–) as no (low quality).

**Figure 2** illustrates the methodological quality of the nine included studies based on QUADAS. **Figure 3** summarizes the risk of bias and adherence of individual studies to these items. **Table 2** provides data on the other nine items of methodological quality. There are two studies has a selection bias (18, 20). For one study (19), performers of ES had received appropriate training, whereas in the other studies, these data were not provided. Four studies (17, 18, 22, 23) were reported to have no commercial funding, but in the other seven studies, there were no data on the source of funding.

**Table 2:** Summary of risk of bias for nine additional items assessing the methodologic quality of the included studies.

First Author [Reference No.]	Thresholds Established?	Technology Unchanged?	Positive Results Defined?	Appropriate Training?	Treatment Withheld?	Observer Variation Reported?	Instrument Variation Reported?	Objectives Prespecified?	Free of Commercial Funding?
Shuzhen (15)	No	Yes	Yes	Unclear	Yes	Unclear	Yes	Yes	Unclear
Yang (16)	Yes	Yes	Yes	Unclear	Unclear	Unclear	Yes	Yes	Unclear
Reginelli (17)	No	Yes	Yes	Unclear	Yes	Unclear	Yes	Yes	Yes
Zhao (18)	Yes	Yes	Yes	Unclear	Yes	Unclear	Yes	Yes	Yes
Moon (19)	Yes	Yes	Yes	Yes	Unclear	Yes	Yes	Yes	Unclear
Wu (20)	No	Yes	Yes	Unclear	Yes	Yes	Yes	Yes	Unclear
Garg (21)	Yes	Yes	Yes	Unclear	Unclear	Unclear	Yes	Yes	Unclear
Shao (22)	No	Yes	Yes	Unclear	Unclear	Yes	Yes	Yes	Yes
Shweel (23)	Yes	Yes	Yes	Unclear	Yes	Unclear	Yes	Yes	Yes

### 3.4 Threshold effects and heterogeneity

The Spearman correlation coefficients were 0.533, -0.214, and -0.500 by heterogeneity analysis (P>0.05), indicating that there was no threshold effect. At the same time, the results of heterogeneity showed that the CUS group (P=0.000, I^2 =^ 79.7%), the ES group (P=0.000, I^2 =^ 95.2%), the combined diagnosis group (P=0.009, I^2 =^ 78.8%). The included literature has large heterogeneity, and the effect size was combined using a random effect model.

### 3.5 Sensitivity analysis

**Figure 4:**
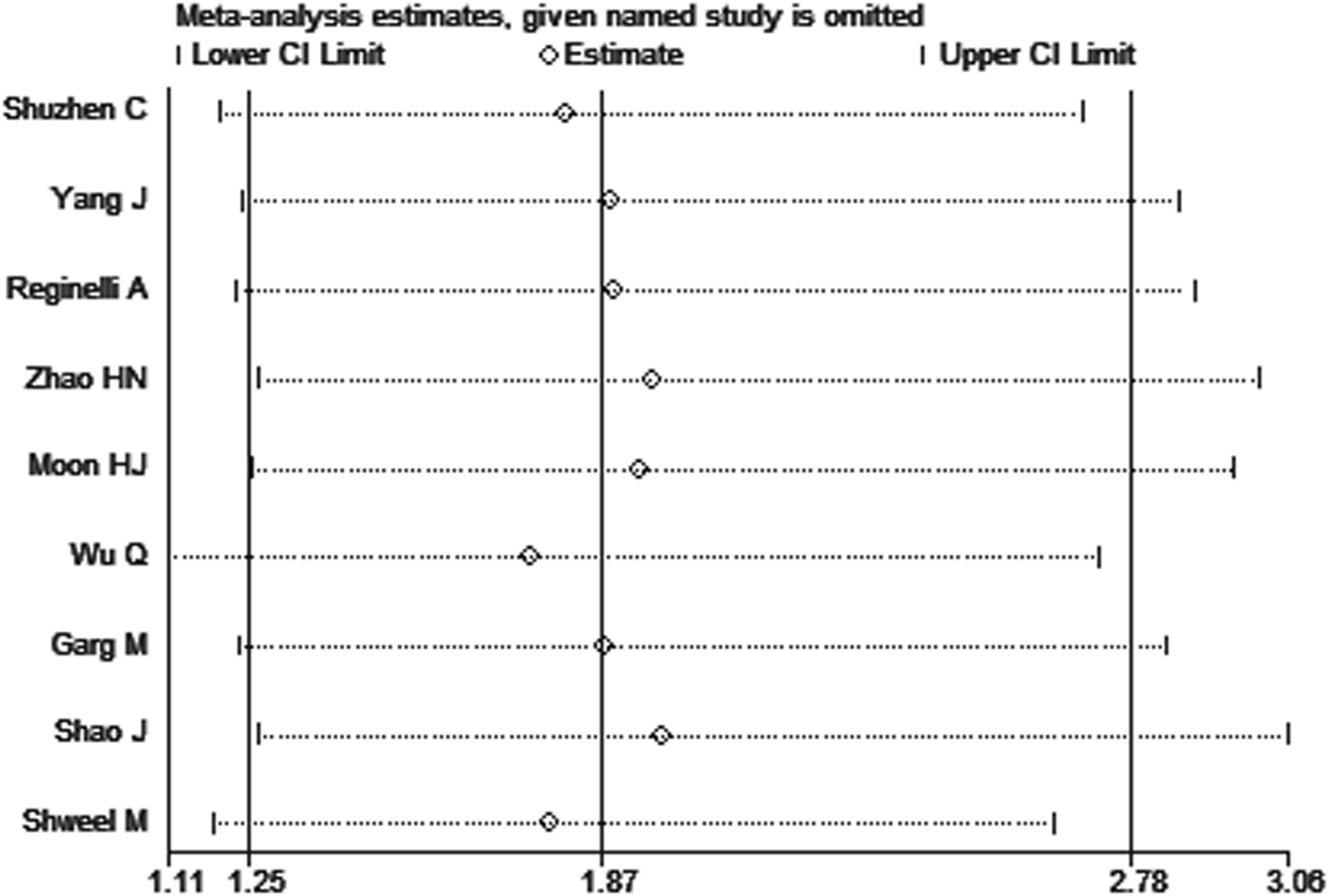
Sensitivity analysis of studies.

To observe the stability of the synthetic results, the data included in the literature were excluded one by one and the sensitivity and specificity were summarized again. It showed that the variables after exclusion were not large, indicating that the stability of the included literature was good and the reliability of the results was high (**Figure 4**).

### 3.6 Meta-regression analysis

As a result of the significant heterogeneity, meta-regression analysis was performed to explore potential sources of heterogeneity. Meta-regression analysis of the CUS group, ES group, and a combined group showed that there was no significant difference between the sources of heterogeneity and the covariates (P>0.05).

### 3.7 Diagnostic accuracy

**Figure 5:**
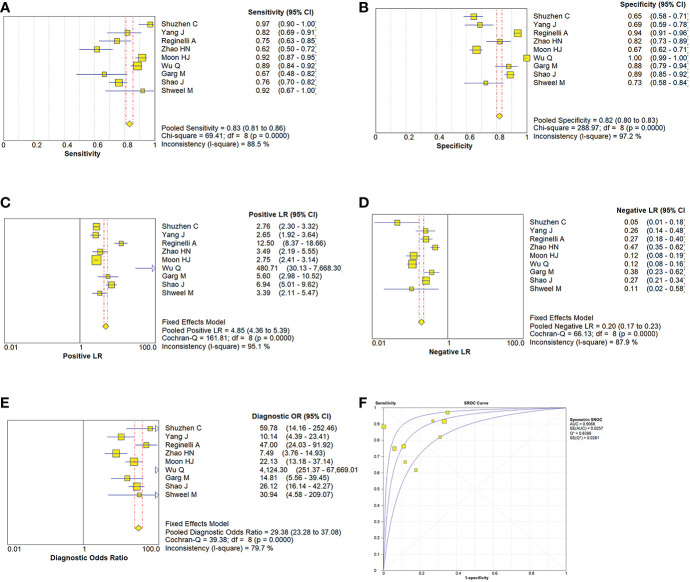
Estimates of conventional ultrasonography assessment for the diagnosis of malignancy thyroid nodules **(A–E)** Forest plots illustrate pooled estimates (diamonds) for sensitivity **(A)**, specificity **(B)**, positive likelihood ratio (LR) **(C)**, negative LR **(D)**, and diagnostic odds ratio **(E) **and corresponding 95% CIs for pooled estimates. **(F)** Summary receiver operating characteristic (SROC) plot for assessing accuracy with corresponding curves indicative of upper and lower bounds of 95% CI. AUC = area under curve, SE = standard error, Q* = summary measure of accuracy derived from the SROC curve.

**Figure 6:**
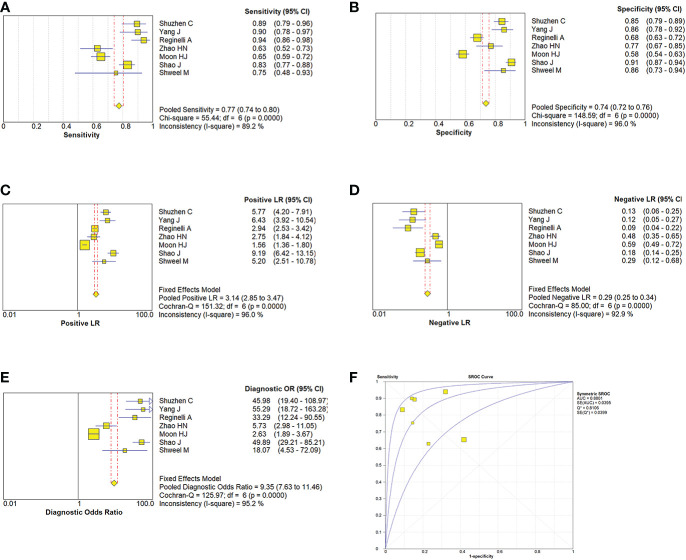
Estimates of elastosonography assessment for the diagnosis of malignancy thyroid nodules. **(A–E)** Forest plots illustrate pooled estimates (diamonds) for sensitivity **(A)**, specificity **(B)**, positive likelihood ratio (LR) **(C)**, negative LR **(D)**, and diagnostic odds ratio **(E)** and corresponding 95% CIs for pooled estimates. **(F)** Summary receiver operating characteristic (SROC) plot for assessing accuracy with corresponding curves indicative of upper and lower bounds of 95% CI. AUC = area under curve, SE = standard error, Q* = summary measure of accuracy derived from the SROC curve.

**Figure 7:**
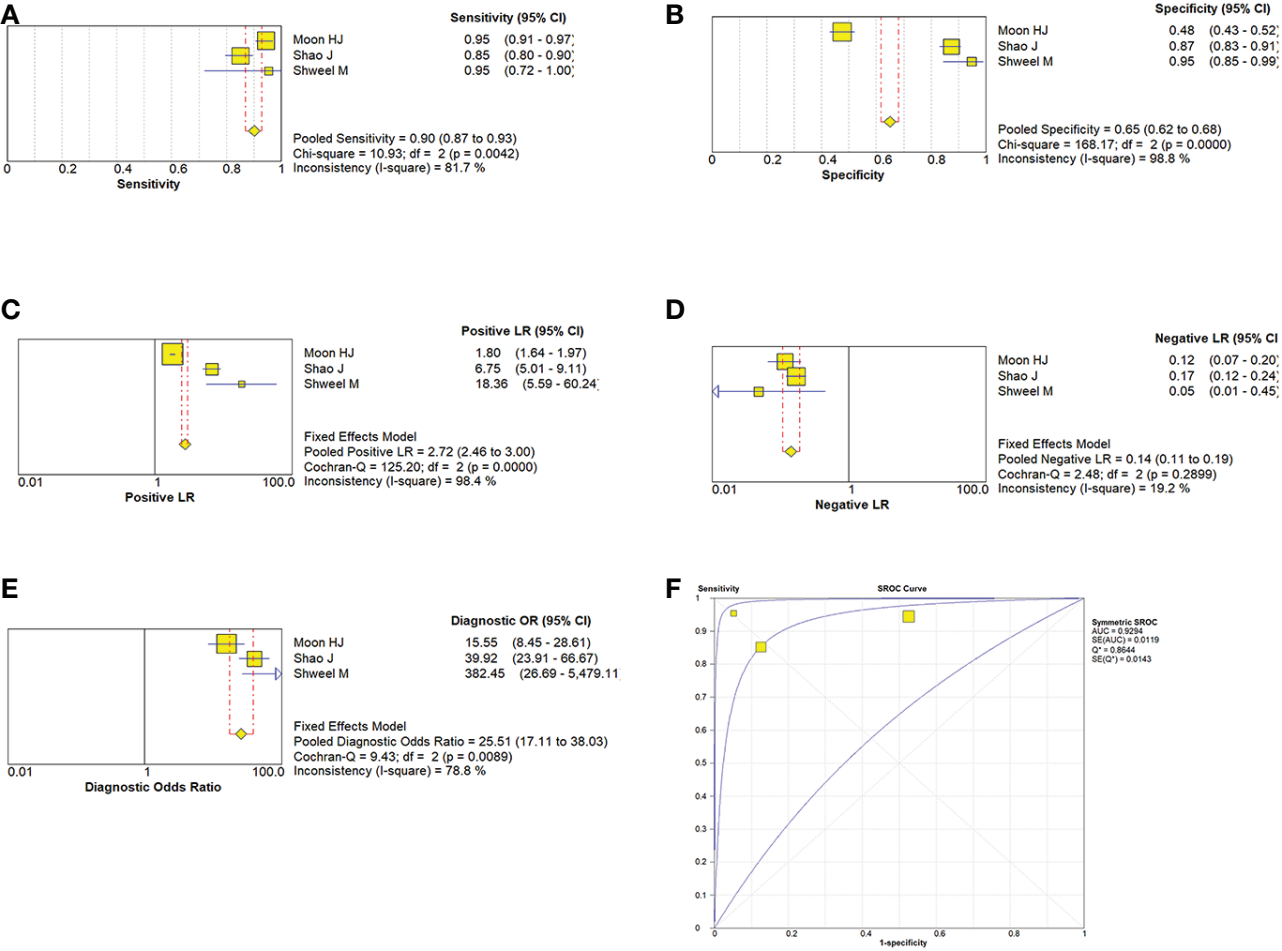
Estimates of the conventional ultrasonography combined with elastosonography for assessment for the diagnosis of thyroid nodules. **(A–E)** Forest plots illustrate pooled estimates (diamonds) for sensitivity **(A)**, specificity **(B)**, positive likelihood ratio (LR) **(C)**, negative LR **(D)**, and diagnostic odds ratio **(E)** and corresponding 95% CIs for pooled estimates. **(F)** Summary receiver operating characteristic (SROC) plot for assessing accuracy with corresponding curves indicative of upper and lower bounds of 95% CI. AUC = area under curve, SE = standard error, Q* = summary measure of accuracy derived from the SROC curve.

Due to the heterogeneity in the calculation of the combined value, the random effect model was used to combine the effect size. The combined sensitivity of CUS, ES, and the combination of the two was 0.833 (95% CI 0.809-0.855), 0.774 (95% CI 0.741-0.804), and 0.902 (95% CI 0.870-0.928), respectively, and the combined specificities were 0.818 (95% CI 0.801-0.835), 0.737 (95% CI 0.715-0.758), 0.649 (95% CI 0.616-0.681), and the areas under the comprehensive ROC curve were 0.9068, 0.8801, and 0.9294, respectively. (**Figures 5**, **6**, **7**). The combined diagnosis group had the highest sensitivity and the lowest negative likelihood ratio, the CUS group had the highest specificity. The diagnostic effect of the combined diagnosis group was the best, followed by the CUS group, and the diagnostic effect of the ES group was relatively poor, as shown in **Table 3**. Therefore, the diagnostic value of ES alone for thyroid malignant nodules is limited.

**Table 3:** Summary of pooled estimates of diagnostic for detecting malignant thyroid nodules.

group	Se	Sp	PLR	NLR	AUC
CUS	0.83	0.82	4.85	0.2	0.9068
ES	0.77	0.74	3.14	0.29	0.8801
Combined CUS and ES	0.9	0.65	2.72	0.14	0.9294

CUS, conventional ultrasound, ES, elastosonography Se, sensitivity; Sp, specificity;

PLR, positive likelihood ratio; NLR, negative likelihood ratio; AUC, area under the curve.

## 4 Discussion

At present, the diagnosis of thyroid nodules mainly relies on ultrasonography, because it is convenient, fast, cheap, and the detection rate of ultrasonography in thyroid diseases is significantly better than that of CT, MRI, and radionuclide, so ultrasonography is the first choice for detection of thyroid diseases (23–25). The malignant characteristics of thyroid nodules may not be obvious in CUS, because when the infiltration scope of the lesions is small or has not broken through the basement membrane, CUS usually shows regular morphology, which is not conducive to diagnosis. ES is based primarily on a comprehensive analysis of tissue hardness for diagnosis, which is different from CUS imaging, so it can better detect small thyroid lesions, and more accurately differentiate between benign and malignant (15, 26). However, the elastic coefficients of various tissues overlap, so there may be misdiagnosis problems in this detection process. Some studies have found that about 30.8% of benign and malignant thyroid nodules have no significant difference in image characteristics (8). Therefore, Fine needle biopsy (FNB) is still the most effective and used method evaluating thyroid nodules (27). However, FNB is invasive and could possibly lead to some complications such as pain, infection and hemorrhage. Thus, an alternative imaging technique providing additional information for identifying thyroid nodules would be greatly valuable. However, there is no consistent conclusion on the accuracy of its diagnosis at present. Based on the summary of existing studies, this study will be systematically and comprehensively evaluating the accuracy, sensitivity, and specificity of CUS combined with ES in the diagnosis of thyroid malignant nodules.

In this study, 9 research papers and 3066 lesions were selected based according to the inclusion criteria. Meta-regression analysis of the three groups showed that there was heterogeneity between the studies. Although the results of the sensitivity analysis found that the meta-analysis results are robust, Meta-regression analysis of the CUS group, ES group, and a combined group showed that there was no significant difference between the sources of heterogeneity and the covariates. We thought that it may be due to the different disease degrees of patients in different studies. Furthermore, the diagnostic criteria of CUS and ES of thyroid nodules in different studies may be inconsistent. The work experience, technique, and machine sensitivity of ultrasound doctors have a certain impact on the diagnosis results, resulting in deviation in interpretation and greater clinical heterogeneity. At the same time, it is difficult to correctly describe the morphology of small nodules with CUS, and the soft and hard conditions of small nodules with ES are easy to overlap with the surrounding normal tissues, resulting in greater heterogeneity in the joint diagnosis group. Since the mean nodule size is not reported in the included literature, it cannot be used for the analysis of covariance. In addition, we only search for English literature and may ignore research or reports in other languages.

In our meta-analysis, the DOR of CUS, ES, and combined group is 29.38, 9.35, and 25.51. DOR reflects the relationship between the results of diagnostic tests and diseases. When the value is>1, the larger the value is, the better the diagnostic test is. This shows that although all three methods are effective, the detection effect of the CUS group and the combined group is better than that of the ES group. However, previous research results on the specificity of ES technology are inconsistent, Rago reported sensitivity of 97% and specificity of 100% for this technique (28), while Tanaka showed sensitivity of 89.1% and specificity of 59.4% (29). We found that the specificity of ES in assessing elasticity score was low, indicating that ES was false positive and not suitable for every patient. We consider these differences for the following reasons: (1) these findings may be misleading and may have been due to a sample selection bias (30), (2) The comparison between ES and CUS in other studys were performed indirectly. (3) malignant thyroid nodules are often combined with other types of benign thyroid diseases. There are more fibrous tissue, calcified tissue, and other components in the lesions of the thyroid gland, so that the elasticity of the nodule is relatively increased or smaller (31). (4) the nodules that grow near the lower level of the thyroid or the isthmus are affected by the surrounding bone. There is a certain error in the detection of tissue impact. (5) Huang (32) found that for some malignant lesions that are too small, the corresponding elastography cannot show the hardness with small differences, which leads to the elastic classification is low, which reduces the detection accuracy.(6) ultrasound elastography cannot be applied to calcified nodules and major cystic lesions, because the ultrasound beam will not pass-through calcification, and the compression of the probe will not lead to tissue strain deformation, so it will produce artifacts in color-coded images, resulting in inaccurate information. (7) ultrasonic elastography is still an unsolved problem in detecting specific types of thyroid cancer. For example, because of its soft texture, unlike other malignant nodules, is easily missed by elastic ultrasound (33). Therefore, only using ES to diagnose thyroid cancer will be misdiagnosed. Therefore, the effect of ultrasonic elastography in differentiating thyroid nodules needs to be further evaluated in future studies.

CUS can supplement other diagnostic evidence, such as irregular shape, hypoechoic, unclear echogenic boundary, and relatively large aspect ratio (≥1), microcalcification and lack of halo, increase the objectivity of diagnosis and reduce misdiagnosis and missed diagnosis (34, 35). Because the imaging manifestations of the masses are closely related to their histopathological characteristics, for some special types of tumors such as follicular thyroid carcinoma, the sonographic images are more common with iso-echoic or slightly hypoechoic parenchymal masses, which are larger and have relatively small calcifications (36). It is unusual and difficult to identify by conventional ultrasonography. In the case of nodular goiter combined with thyroid cancer, the softness and hardness of ultrasonic elastography techniques overlap, so the combined application of CUS and ES is particularly necessary. When ES was Combined with CUS, the sensitivity to differentiate malignant thyroid nodules was 90%, the positive likelihood ratio was 2.72, the negative likelihood ratio was 0.14, and the corresponding area under the curve was 0.9294, indicating that the detection rate of the combined diagnosis of malignant thyroid nodules was the highest.

This meta-analysis has some limitations, which should be taken into account while interpreting the conclusions. Firstly, in order to determine which imaging modality is superior, rigorous research should be carried out adopting these two ultrasound technologies on the same cohort of patients. We have carried out strict procedures to review articles, so there are few qualified studies that meet the inclusion criteria. Secondly, there may be English language bias, because we only include English publications. Finally, although meta-regression excluded the influence of study design, region, and method, other factors such as ultrasound equipment, threshold values, index of elastography, and demographic characteristics would like to be taken into account. Due to the limited included studies, we were not able to perform meaningful subgroups on the basis of other factors mentioned above. Hence, more rigorous studies in the future are needed to address these methodological limitations.

In conclusion, our latest research shows that ES revealed a limited value for diagnosing malignant thyroid nodules, which cannot replace CUS. It can be employed as an auxiliary tool of conventional ultrasound and may reduce unnecessary fine needle biopsy.

## Data availability statement

The original contributions presented in the study are included in the article/supplementary material. Further inquiries can be directed to the corresponding author.

## Author contributions

DZ: Software, Writing - original draft. X-NW: Data curation. LJ: Visualization, Investigation. C-XY: Visualization, Investigation. Y-NC: Writing - review and editing. X-JY: Supervision. M-FP: Conceptualization, Methodology, Supervision. All authors contributed to the article and approved the submitted version.
